# Mallory-Weiss tear with intestinal obstruction: case report and literature review

**DOI:** 10.1093/jscr/rjag585

**Published:** 2026-07-23

**Authors:** Dana T Gharib, Omer H Ghalib, Sivan H Salih, Soran H Tahir, Kayhan A Najar, Hoshmand R Asaad, Karokh F Hamahussein, Rahand A Yousif, Berun A Abdalla, Fahmi H Kakamad

**Affiliations:** Department of Gastroenterology, Gastroenterology and Hepatology Teaching Hospital, Zanko Street, Sulaymaniyah 46001, Iraq; Department of Gastroenterology and Hepatology, Smart Health Tower, Madam Mitterrand Street, Sulaymaniyah 46001, Iraq; Department of Gastrointestinal Surgery, Smart Health Tower, Madam Mitterrand Street, Sulaymaniyah 46001, Iraq; Department of Gastroenterology and Hepatology, Smart Health Tower, Madam Mitterrand Street, Sulaymaniyah 46001, Iraq; Department of Radiology, Smart Health Tower, Madam Mitterrand Street, Sulaymaniyah 46001, Iraq; Department of Gastroenterology and Hepatology, Smart Health Tower, Madam Mitterrand Street, Sulaymaniyah 46001, Iraq; Kscien Organization, Hamdi Street, Azadi Mall, Sulaymaniyah 46001, Kurdistan, Iraq; Department of Gastroenterology, Gastroenterology and Hepatology Teaching Hospital, Zanko Street, Sulaymaniyah 46001, Iraq; Department of Gastroenterology and Hepatology, Smart Health Tower, Madam Mitterrand Street, Sulaymaniyah 46001, Iraq; Department of Gastroenterology and Hepatology, Smart Health Tower, Madam Mitterrand Street, Sulaymaniyah 46001, Iraq; Department of Scientific Affairs, Smart Health Tower, Madam Mitterrand Street, Sulaymaniyah 46001, Iraq; Department of Medical-Surgical Ward, Smart Health Tower, Madam Mitterrand Street, Sulaymaniyah 46001, Iraq; Kscien Organization, Hamdi Street, Azadi Mall, Sulaymaniyah 46001, Kurdistan, Iraq; Department of Scientific Affairs, Smart Health Tower, Madam Mitterrand Street, Sulaymaniyah 46001, Iraq; Kscien Organization, Hamdi Street, Azadi Mall, Sulaymaniyah 46001, Kurdistan, Iraq; Department of Scientific Affairs, Smart Health Tower, Madam Mitterrand Street, Sulaymaniyah 46001, Iraq; College of Medicine, University of Sulaimani, Madam Mitterrand Street, Sulaymaniyah 46001, Kurdistan, Iraq; Department of Thoracic and Vascular Surgery, Smart Health Tower, Madam Mitterrand Street, Sulaymaniyah 46001, Iraq

**Keywords:** Mallory-Weiss tear, small bowel obstruction, upper gastrointestinal bleeding, distal small intestine obstruction, hematemesis

## Abstract

Mallory-Weiss tear (MWT) is a mucosal laceration at the esophagogastric junction. This case report aims to present a rare case of MWT associated with small bowel obstruction (SBO). A 74-year-old female presented with hematemesis and abdominal pain. Esophagogastroduodenoscopy (EGD) showed blood in the esophagus and stomach with a tear in the cardia. Computed tomography scan of the abdomen demonstrated multiple dilated small bowel loops with a distal ileal transition point in the pelvis. The patient underwent a midline laparotomy. Seven cases of MWT and SBO were identified. The most common presentation was hematemesis, seen in six patients (85.71%). Abdominal pain was reported in one case (14.28%). Two patients were treated with medication (28.57%), and five underwent surgery (71.42%). This study highlights the importance of recognizing MWT not merely as an isolated cause of upper gastrointestinal bleeding, but potentially as a secondary manifestation of increased intra-abdominal pressure.

## Introduction

Mallory-Weiss tear (MWT) is a medical condition characterized by mucosal laceration at the esophagogastric junction, accounting for 3%–10% of all upper gastrointestinal bleeding (UGIB) cases [1, 2]. Mallory-Weiss tear usually results from prolonged vomiting, coughing, straining, or increases in intra-abdominal pressure. Additional risk factors include bulimia nervosa and gastroesophageal reflux disease. Excessive alcohol consumption is a major predisposing factor [1]. It predominantly affects younger individuals and males [2]. Mallory-Weiss tear can also be due to other pathologies. Findings that suggest other life-threatening underlying diseases, such as intestinal obstruction, should be investigated. Mallory-Weiss tear presenting with small bowel obstruction is uncommon [3].

This study aims to report a rare case of MWT associated with small bowel obstruction (SBO), with limited available data on it. The report was prepared in accordance with the CaReL guidelines, and all referenced sources were critically assessed for reliability [4, 5].

## Case presentation

### Patient information

A 74-year-old female presented with hematemesis and abdominal pain for 2 days. She had a history of ischemic heart disease (IHD) and was on a beta-blocker and clopidogrel oral medications. Surgical history included cholecystectomy and hysterectomy.

### Clinical findings

The patient was alert, oriented, and hemodynamically stable.

### Diagnostic assessments

Laboratory investigations showed mild leukocytes 11.7 × 10^9^/L (normal range: 3.4–9.6 × 10^9^/L) and C-reactive protein 13.3 mg/L (normal range: below 3.0 mg/L), indicating an underlying inflammation (Table 1). Esophagogastroduodenoscopy (EGD) showed blood in the esophagus and stomach with a tear in the cardia, while full assessment was limited by patient irritability (Fig. 1). Abdominal US (ultrasonography) demonstrated multiple dilated fluid-filled bowel loops with sluggish peristalsis and free fluid. Subsequently, a computed tomography (CT) scan of the abdomen with oral and intravenous contrast demonstrated multiple dilated small bowel loops with a distal ileal transition point in the pelvis, mild terminal ileal wall thickening, and mild ascites, without definite evidence of bowel ischemia, suggestive of adhesive SBO (Fig. 2).

**Table 1:** Summary of the findings of the laboratory tests performed for the patient.

Test category	Result	Normal range
WBC count	11.7 × 10^9^/L	3.4–9.6 × 10^9^/L
granulocytes	80.6%	40%–75%
HGB	9.9 g/dL	12.3–15.3 gm/dL
HCT	30.00%	35.5%–44.9%
RBC	3.04 × 10^12^/L	3.92–5.13 × 10^12^/L
platelets	180 × 10^9^/L	157–371 × 10^9^/L
glucose	142 mg/dL	70 and 140 mg/dL
AST	35 U/L	8–43 U/L
ALT	18 U/L	7–45 U/L
amylase	92 U/L	30–110 U/L
lipase	32 U/L	0–160 U/L
urea	55.4 mg/dL	6–21 mg/dL
creatinine	0.69 mg/dL	0.6–1.1 mg/dL
CRP	13.3 mg/L	below 3.0 mg/L
calcium	7.9 mg/dL	8.6–10.2 mg/dL
sodium	141.1 mmol/L	135–145 mmol/L
potassium	3.88 mmol/L	3.5–5.0 mmol/L
chloride	103.1 mmol/L	98–106 mmol/L
PT	17.1 s	11.0–13.5 s
INR	1.32	0.8–1.1
PTT	35.4 s	25–35 s
TSH	0.47 μIU/mL	0.4–5.0 μIU/mL
FT3	1.30 pmol/L	3.1–6.8 pmol/L
FT4	10.26 pmol/L	10.3–23.2 pmol/L
Vitamin B12	110.4 pg/mL	200–900 pg/mL

WBC: White blood cell, HGB: Hemoglobin, HCT: Hematocrit, RBC: Red blood cell, AST: Aspartate aminotransferase, ALT: Alanine aminotransferase, CRP: C-reactive protein, PT: Prothrombin time, INR: International normalized ratio, PTT: Partial thromboplastin time, TSH: Thyroid-stimulating hormone, FT3: Free triiodothyronine, FT4: Free thyroxine.

**Figure 1:**
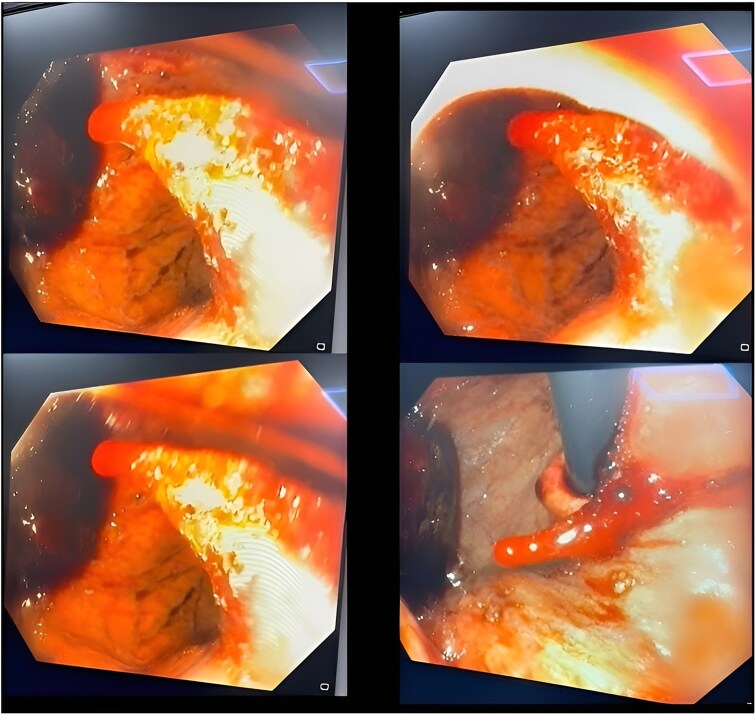
Esophagogastroduodenoscopy. A longitudinal mucosal tear is seen at the esophagogastric junction, associated with adherent clot and slight oozing. The surrounding mucosa appears congested and hemorrhagic, with retained altered blood in the stomach. The appearance is consistent with Mallory-Weiss tear with stigmata of active recent bleeding.

**Figure 2:**
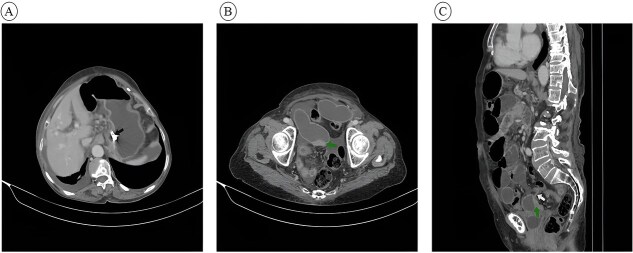
CT abdomen with intravenous contrast. (A) Upper abdomen axial section shows a clip applied in the cardia region of the stomach to stop upper gastrointestinal bleeding (black arrow). (B) The axial section of the pelvis region shows the small intestinal obstruction transition point. (C) Sagittal section shows the transition point of small intestinal obstruction (green arrow) with a band at the site of obstruction (white arrow).

### Therapeutic intervention

The patient was admitted, and high-dose esomeprazole was initiated. Blood preparation and repeat EGD within 24–48 h were recommended. She had gastrointestinal bleeding, considered secondary to the SBO rather than solely the cardia tear. A midline laparotomy was performed. The peritoneal cavity contained ~400 mL of free reactive fluid, with no pus or blood. Intraoperatively, the small bowel was dilated due to terminal ileal obstruction from a compressing adhesive band arising from a previous hysterectomy. The affected small bowel segment was ischemic with multiple patches of gangrene, but without perforation. Segmental resection with enterostomy was performed. The patient remained in the intensive care unit for the first 3 days due to hypotension requiring noradrenaline support. Oral intake was started after 3 days.

### Follow-up and outcomes

The patient improved clinically. She was discharged 7 days after surgery on her cardiac medications.

## Discussion

Mallory-Weiss tear is a recognized cause of nonvariceal UGIB. It was first described by Kenneth Mallory and Soma Weiss in 1929. Although it typically presents as a single longitudinal mucosal tear, multiple or circumferential lesions may occur. Most lacerations are confined to the mucosa; however, deeper extension can involve blood vessels and result in significant bleeding [2]. While MWT often heal on their own, in severe cases, medical intervention might be necessary to prevent complications such as bleeding, infection, or perforation of the esophagus or stomach. The precise mechanism of MWT remains incompletely defined but includes a complex mix of mechanical and mucosal factors [2]. Small bowel obstruction is a common surgical emergency, accounting for nearly 20% of hospital admissions for acute abdominal pain [6].

A targeted review of relevant literature was conducted. Studies were included in the literature review based on their relevance. A total of seven previously reported cases from 1994 to 2024 of MWT and SBO were identified in the literature. Three patients were females, and four were males, with a mean age of ~53 years (range: 15–77 years). The most prevalent presentation was hematemesis (6/7, 85.71%). Abdominal pain was reported in one (5/7, 71.43%), US (2/7, 28.57%), and CT scan (2/7, 28.57%). Management varied; three patients were treated non-operatively (42.86%), and four underwent surgery (57.14%). Follow-ups were mixed, postoperative stability was reported in five cases (71.42%), long-term recovery was mentioned in one case (14.28%), and one patient died postoperatively due to multiorgan failure (14.28%) [1, 3, 7–11] (Table 2).

**Table 2:** Summary of Mallory–Weiss tear and small bowel obstruction reported cases.

Author/Reference	Year	No. of patients	Sex	Age	Presentation	Diagnostic technique	Management	Follow-up
Gupta *et al.* [1]	2023	1	F	77	Hematemesis.	X-ray, US, EGD	Medication (Pantoprazole, Sucralfate)	Hemodynamically stable during discharge, review in the OPD after 10 days.
Jane *et al.* [11]	2024	1	M	15	Abdominal pain and hematemesis.	US, EGD	Pantoprazole, clips were applied to the Mallory-Weiss tear	Stable, eating and drinking well upon discharge.
Scallion & Wei [3]	1994	1	M	40	Melena, abdominal pain and tenderness, nausea, and hematemesis.	X-ray, EGD	Laparotomy	Dies on the 3^rd^ day post op due to multiple organ failure
Sumalatha *et al.* [7]	2023	1	M	67	Hematemesis, heaviness in chest associated with back pain, sweating, SOB, giddiness, and vomiting	EGD	Endoscopic Sclerotherapy and clipping of the bleeding ulcer with metal clips were done	On regular follow up and is doing better to date (28 months) with noticeable improvement in quality of life.
Branch *et al.* [10]	2024	1	F	49	abdominal pain, dark, loose stools, and hematemesis.	CT angiography	A Graham patch repair was performed. Resection of the jejunojejunostomy intussusception and ischemic bowel was then performed. Reconstruction was performed, and then anastomosis	The patient recovered well, was advanced to a pureed diet after return of bowel function and discharged home 4-days after the operation.
Wong *et al.* [8]	2022	1	F	62	Melena, abdominal pain, and hematemesis	X-ray, EGD	exploratory laparotomy. A Graham patch repair was performed on the jejunal side of the gastrojejunal anastomosis. Resection of the jejunojejunostomy intussusception and ischemic bowel was then performed	Stable. Followed up in the clinic without any further symptoms.
Tepelenis *et al.* [9]	2021	1	M	59	Abdominal pain	CT scan	exploratory laparotomy via a midline incision, the band was ligated and divided	The patient recovered uneventfully, discharged after 3 days.

M: Male, F: Female, EGD: Esophagogastroduodenoscopy, CT: Computed Tomography, US: Ultrasonography, SOB: Shortness of breath, TPN: Total Parenteral Nutrition, NA: Non-applicable.

Clinical manifestations of MWT include hematemesis, with melena and hematochezia; pain is uncommon unless complications occur [2, 7]. Patients with SBO typically present with acute, crampy abdominal pain and vomiting, [6, 11]. Scallion and Wei described that when severe abdominal pain is continuously present, progression of the obstruction to intestinal strangulation and gangrene should be suspected [3]. In this case, hematemesis and abdominal pain were attributed to MWT and SBO.

The diagnosis of MWT should be considered in patients with UGIB preceded by vomiting and confirmed by EGD, which enables direct visualization, hemostasis, exclusion of alternative sources, and assessment of bleeding activity [2, 8]. Small bowel obstruction diagnosis relies on laboratory and imaging studies. Laboratory evaluation includes complete blood count, electrolytes, and renal function. Schick et al. reported the CT scan of the abdomen to be the gold standard [6], while US provides a rapid, noninvasive assessment of bowel distension, peristalsis, and free fluid [6, 11]. In this case, EGD revealed a tear in the cardia with UGIB; laboratory studies were comprehensive, and abdominal US showed dilated loops with sluggish motility and free fluid. Although CT did not detect the bowel ischemia and gangrene found intraoperatively, the findings emphasize the diagnostic value of early clinical and ultrasonographic assessment.

Management of MWT is typically conservative, as most cases resolve spontaneously with proton pump inhibitors, while active bleeding requires endoscopic hemostasis with success rates exceeding 90% [2]. In contrast, the optimal management of SBO remains debated. Scallion and Wei observed that surgery is the preferred way to correct the underlying mechanical condition for intestinal obstruction [3]. Although nonoperative management is recommended for uncomplicated cases, delayed surgery increases the risk of ischemia and gangrene [6, 11]. In this case, conservative therapy was adequate for MWT, whereas early surgical intervention for SBO was required because of ischemia and gangrene. Notably, although CT is the diagnostic gold standard, it failed to detect ischemia, highlighting concerns about its sensitivity and emphasizing the importance of integrating clinical and ultrasonographic findings for early detection of bowel compromise.

Postoperative care includes monitoring for complications such as infection, anastomotic leak, or obstruction. Preventive strategies to reduce SBO recurrence, such as minimally invasive techniques and adhesion barriers, remain subjects of debate [6]. In this case, no recurrence of MWT or SBO was observed.

Mallory-Weiss tear typically has a favorable prognosis [2]. Conversely, SBO outcomes depend on the underlying cause and the timeliness of intervention: simple cases have low mortality (1%–5%), whereas complicated SBO carries a higher mortality rate (10%–40%) [6].

Our study has several limitations. First, the initial upper EGD was limited by patient irritability. Second, the preoperative CT scan imaging failed to identify the bowel ischemia and gangrene found intraoperatively. Finally, due to the patient’s emergent surgical intervention, the repeat EGD to confirm the healing of the cardia tear was deferred postoperatively.

## Conclusion

This study highlights the importance of recognizing MWT not merely as an isolated cause of UGIB, but potentially as a secondary manifestation of increased intra-abdominal pressure. High suspicion must be maintained for underlying SBO when hematemesis is preceded by forceful, repeated vomiting.

## Data Availability

The datasets used and/or analyzed during the current study are available from the corresponding author upon reasonable request.
